# Detection of juvenile hormone agonists by a new reporter gene assay using yeast expressing *Drosophila* methoprene‐tolerant

**DOI:** 10.1002/2211-5463.13277

**Published:** 2021-08-30

**Authors:** Sayoko Ito‐Harashima, Mai Matsuura, Eiji Takada, Masanobu Kawanishi, Yoshiaki Nakagawa, Takashi Yagi

**Affiliations:** ^1^ Department of Biological Science Graduate School of Science Osaka Prefecture University Sakai Japan; ^2^ Division of Applied Science Graduate School of Agriculture Kyoto University Sakyo‐ku Japan

**Keywords:** juvenile hormone, juvenile hormone agonist, methoprene‐tolerant, insect growth regulator, yeast reporter gene assay

## Abstract

Juvenile hormones (JHs) are sesquiterpenoids that play important roles in the regulation of growth, metamorphosis, and reproduction in insects. Synthetic JH agonists (JHAs) have been used as insecticides and are categorized as a class of insect growth regulators (IGRs). Natural JHs and synthetic JHAs bind to the JH receptor methoprene‐tolerant (Met), which forms a functional JH‐receptor complex with steroid receptor coactivators, such as *Drosophila melanogaster* Taiman (Tai). The ligand‐bound Met–Tai complex induces the transcription of JH response genes by binding to specific DNA elements referred to as JH response elements (JHREs). In the present study, we established a reporter gene assay (RGA) for detecting natural JHs and synthetic JHAs in a yeast strain expressing *D. melanogaster* Met and Tai. The yeast RGA system detected various juvenoid ligands in a dose‐dependent manner. The rank order of the ligand potencies of the juvenoids examined in the yeast RGA linearly correlated with those of RGAs for Met–Tai established in mammalian and insect cells. Our new yeast RGA is rapid, easy to handle, cost‐effective, and valuable for screening novel JHAs.

## Graphical abstract

Synthetic juvenile hormone agonists (JHAs) that have been used as insecticides mimic the action of insect JHs and disrupt signaling *via* the JH‐receptor methoprene‐tolerant (Met). In this study, we developed a novel reporter gene assay to measure ligand activity of natural JHs and synthetic JHAs by co‐expressing *Drosophila melanogaster* Met and steroid receptor coactivator Taiman in yeast *Saccharomyces* *cerevisiae*.

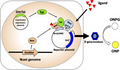

**Abbreviations**
20E: 20‐hydroxyecdysone
AhR: aryl hydrocarbon receptor
Arnt: aryl hydrocarbon receptor nuclear translocator
bHLH‐PAS: basic helix‐loop‐helix Per/Arnt/Sim
DMSO: dimethyl sulfoxide
DTT: dithiothreitol
EcR: ecdysone receptor
EcRE: ecdysone response element
Gce: germ cell‐expressed
IGR: insect growth regulator
JH: juvenile hormone
JHA: juvenile hormone agonist
JHR: juvenile hormone receptor
JHRE: juvenile hormone response element
JHR: juvenile hormone receptor
*Kr‐h1*
: *Krüppel homolog‐1*
Met: methoprene‐tolerant
MH: molting hormone
ONP: *o‐*nitrophenol
ONPG: *o‐*nitrophenyl‐β‐D‐galactopyranoside
RGA: reporter gene assay
Tai: Taiman
USP: ultraspiracle

Juvenile hormones (JHs) are sesquiterpenoid compounds that regulate the endocrine system in insects, in conjunction with molting hormones (MHs) with steroid backbones. These two types of hormones are coordinately involved in various physiological processes in insects, such as growth, metamorphosis, and reproduction [1, 2, 3, 4, 5, 6, 7, 8, 9, 10]. Synthetic chemicals with JH activities, JH agonists (JHAs), have been developed as insecticides. JHAs are categorized as insect growth regulators (IGRs) and are considered to be useful for controlling insect pests because they interfere with insect‐specific hormone responses [11, 12, 13, 14, 15].

The JH receptor gene *Met* was identified as the gene responsible for the methoprene‐resistant phenotype of a *Drosophila melanogaster* mutant, which encodes the ligand‐dependent transcription factor of the basic‐helix‐loop‐helix Per/Arnt Sim (bHLH‐PAS) family [16, 17]. A genome‐wide survey of factors containing the bHLH domain revealed the conservation of the paralogous gene germ cell‐expressed (*Gce*) in *Drosophila* species [18, 19]. It was shown that Gce had a partly conserved function with Met in transducing JH action [20, 21]. Further study demonstrated that JH‐binding capacity of Met and Gce were required to mediate JH action during normal development of *D*. *melanogaster*, establishing Met/Gce as JH receptors *in vivo* [22]. Ligand‐bound Met/Gce proteins heterodimerize with other bHLH‐PAS family proteins, such as *D. melanogaster* Taiman (Tai) [23, 24]. The complex of JH‐bound Met and Tai binds to specific *cis*‐acting DNA sequences referred to as JH response elements (JHREs) in order to induce the transcription of early JH response genes [23, 24, 25]. The anti‐metamorphic gene *Krüppel homolog 1* (*Kr‐h1*) was identified as a target of Met, and a 13‐nucleotide motif containing an E‐box (CACGTG) as JHRE. The DNA motif was essential for the binding of Met–Tai to induce the transcriptional activation of *Kr‐h1* [26, 27, 28].

The activities of natural JHs and synthetic JHAs have traditionally been examined using whole body [29, 30, 31, 32, 33] or cultured insect cell lines to detect JH‐dependent morphological changes [34, 35, 36]. Since the cloning of *Met*/*Gce* genes, *in vitro* ligand‐receptor binding assay procedures have been established [17, 22, 37, 38]. A reporter gene assay (RGA) is an alternative procedure for examining the JH‐like activity of synthetic chemicals. In RGA systems, the JH‐dependent activation of Met‐mediated gene expression is monitored using reporter gene constructs under the control of JHRE. The two‐hybrid‐based method to detect JH‐dependent interactions between Met and Tai is also employed. To date, a number of RGAs for Met have been established in insect and mammalian cells [39]. We previously established yeast RGAs for the ligand‐dependent transcription factors of animals, such as mammalian aryl hydrocarbon receptors (AhRs)–AhR nuclear translocators (Arnts) in bHLH‐PAS family proteins [40, 41] and human steroid hormone receptors and insect ecdysone receptors (EcRs)–ultraspiracles (USPs) in the nuclear receptor superfamily [42, 43, 44]). These yeast RGAs are simpler, easier to handle, and more cost‐effective than mammalian or insect cell‐based bioassays and instrumental analyses.

In the present study, we established a novel yeast RGA to quantitatively measure the activities of JHs and JHAs in a recombinant yeast strain. Met and Tai from *D. melanogaster* were expressed in yeast cells carrying the *lacZ* reporter plasmid, with JHRE being identified in the *Bombyx mori Kr‐h1* gene [26] (Fig. 1). The ligand responses of the yeast RGA were examined using various juvenoids, and its sensitivity was compared with those of previously established mammalian or insect cell RGAs for Met–Tai.

**Fig. 1:**
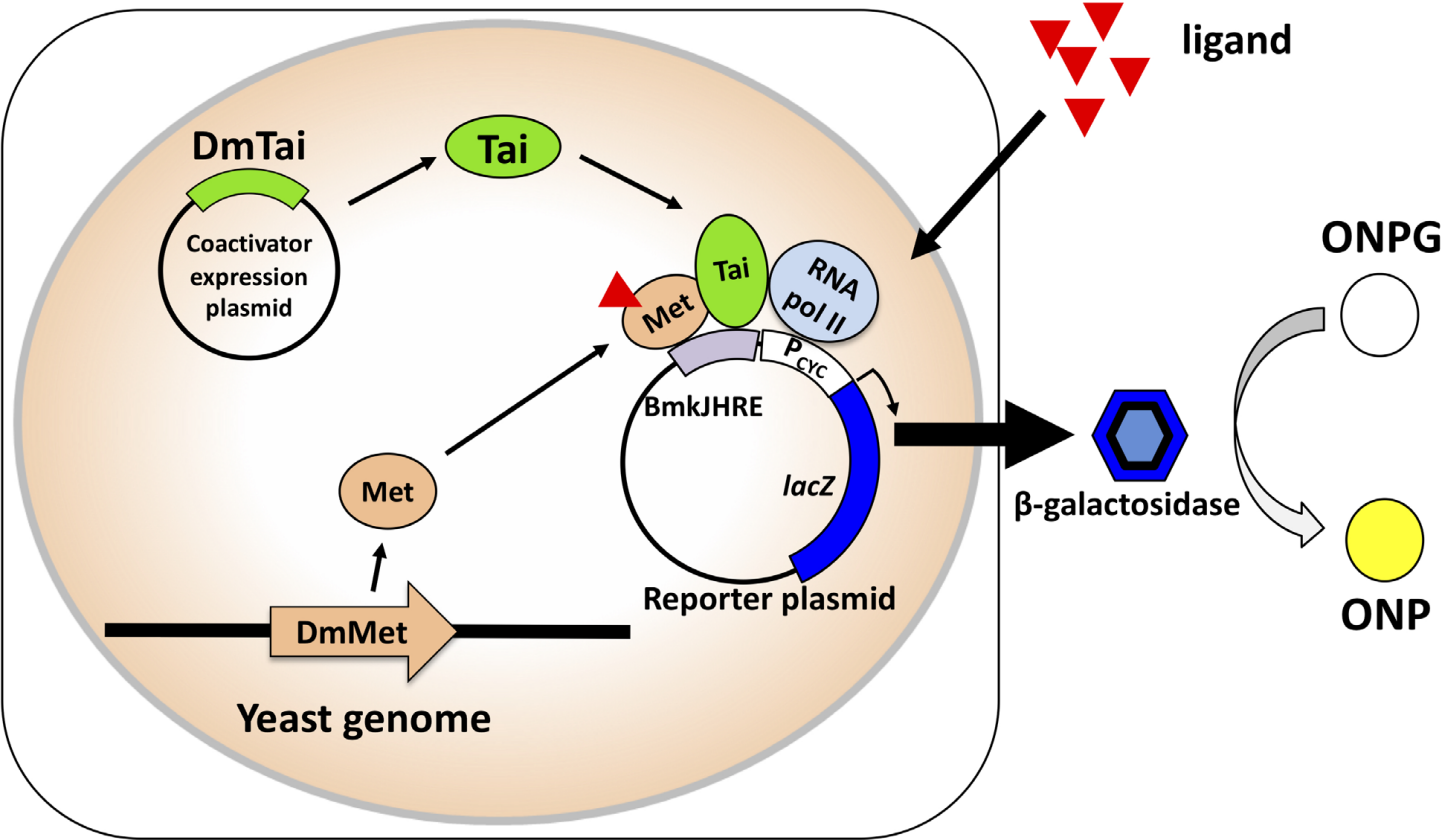
Principle of the yeast RGA for *D. melanogaster* Met. The expression plasmid for the juvenile hormone receptor Met was integrated into the yeast genome by homologous recombination. The reporter plasmid and Tai expression plasmid were maintained as circular plasmids. In response to juvenoids, Met and the steroid receptor coactivator Tai form a heterodimer and bind to the upstream response elements (BmkJHRE) of the *lacZ* reporter gene in yeast cells. The ligand‐bound Met–Tai complex recruits the RNA pol II general transcription factor of yeast and induces the expression of β‐galactosidase. The expression of β‐gal may be visualized by the development of a yellow color due to the accumulation of ONP in the assay buffer.

## Methods

### Strains and media

The *Escherichia coli* strain DH5α was used as a host to amplify plasmid DNA. *Saccharomyces cerevisiae* W303a (*MAT*a, *ura3‐1*, *ade2‐1*, *trp1‐1*, *leu2‐3*, *his3‐11*, *15*, *can1‐100*) was used to establish RGA. Yeast extract peptone dextrose (YPD) and synthetic dextrose complete dropout (SDC‐X) media were prepared as previously described [45]. Synthetic galactose complete dropout (SGC‐X) media contained 2% (w/v) galactose instead of dextrose. All solid media contained 2% (w/v) agar in plates.

### Chemicals

Dimethyl sulfoxide (DMSO) and dithiothreitol (DTT) were obtained from Nacalai Tesque (Kyoto, Japan). JH III, (*S*)‐hydroprene, methoprene, pyriproxyfen, and *o‐*nitrophenyl‐β‐D‐galactopyranoside (ONPG) were purchased from Sigma‐Aldrich Chemical Co. (St. Louis, MO, USA). Methyl farnesoate was obtained from Echelon Biosciences Inc. (Salt Lake City, UT, USA). Fenoxycarb was supplied from Santa Cruz Biotechnology, Inc. (Dallas, TX, USA). JH 0, I, and II were synthesized as previously described [46]. Restriction enzymes, DNA modification enzymes, and other chemicals were obtained from Wako Pure Chemical Industries, Ltd. (Osaka Japan), Nacalai Tesque, Sigma‐Aldrich Chemical Co., TaKaRa Bio Inc. (Otsu, Japan), or TOYOBO Co., Ltd. (Osaka, Japan).

### Plasmid construction

The *D. melanogaster* Met expression plasmid and reporter plasmid carrying the JHRE identified in the *B*. *mori Kr‐h1* gene promoter [26] were constructed to develop the Met ligand assay yeast strain. The primers used in the present study were synthesized by Sigma‐Aldrich Japan (Tokyo, Japan) and are listed in Table S1.

DNA fragments containing the Met (DDBJ/EMBL/GenBank accession number NM_078571) open reading frame (ORF) were obtained from *D. melanogaster* larva poly A^+^ RNA (Clontech, Palo Alto, CA, USA) using a reverse‐transcription polymerase chain reaction (RT‐PCR). First, cDNA was obtained using SuperScript III reverse transcriptase (Invitrogen, Carlsbad, CA, USA). Met cDNA was amplified by PCR with the primer pair DmMet Fwd and DmMet Rev, which contained a restriction site and/or yeast ribosomal binding consensus sequence near the initiation codon. PCR was performed using high‐fidelity PCR polymerase KOD‐plus‐ver. 2 (TOYOBO Co., Ltd.) according to the manufacturer’s instructions. Amplified fragments were digested with *Sma*I and *Eco*RI, and cloned into the corresponding sites of multicloning site (MCS) 2 of the expression vector pUdp6 [47]. Plasmids were isolated and purified using a QIAGEN Mini Prep Kit (Valencia, CA, USA), and the nucleotide sequences of Met ORF were confirmed using the ABI DNA sequencer. A sequence analysis was performed using the Genetyx‐Mac ver. 13.0.3 and Genetyx‐Mac/ATSQ ver. 4.2.4 programs (Genetyx Corporation, Tokyo, Japan).

We found a sequence alteration in the cloned Met ORF, an A to C alteration at nucleotide position 307, which was associated with an amino acid substitution, threonine (ACC) to proline (CCC) at codon 103. Therefore, Met ORF was amplified again from cDNA by PCR using KOD‐plus‐ver. 2 in three independent experiments and then purified and sequenced. Codon 103 of Met was reconfirmed as the CCC encoding proline in all three independently amplified DNA fragments. The T103P alteration in our Met clone appeared to be polymorphism [48]. Since the plasmid was functional in terms of the JH/JHA‐dependent induction of reporter genes in yeast cells, it was designated as pUdp6‐DmMet and used to establish the yeast strain for the Met–Tai assay in the present study.

To construct the reporter plasmids carrying JHRE of *B*. *mori Kr‐h1* (BmkJHRE), one or two copies of oligonucleotides containing these sequences were inserted into the MCS of the pRW95‐3 vector [49], upstream of the CYC minimal promoter. The oligonucleotides Bm‐kJHRE Fwd and Bm‐kJHRE Rev were annealed, phosphorylated with T4 polynucleotide kinase, ligated, and inserted into the *Spe* I site of pRW95‐3. The copy numbers and orientation of the oligonucleotides on each plasmid were confirmed by sequencing. The resultant plasmids were designated as pYTβ‐BmkJHRE × 1 and pYTβ‐BmkJHRE × 2.

The plasmid pESC‐Leu‐DmTai for the expression of Tai was constructed in our previous study [44]. Briefly, Tai ORF amplified from *D. melanogaster* larva poly‐A^+^ RNA by RT‐PCR was cloned into MCS 2 on the yeast expression vector pESC‐Leu (Agilent Technologies, Inc., Santa Clara, CA, USA).

### Establishment of the yeast RGA

Yeast transformation was performed using the lithium acetate procedure as previously described [50]. To develop a yeast RGA for Met–Tai, one of the reporter plasmids and the expression plasmid for Tai, pESC‐Leu‐DmTai, were introduced into the wild‐type yeast strain W303a. A transformant grown on SDC‐TRP/LEU agar medium was isolated and used as a host for subsequent transformation. The Met expression plasmid pUdp6‐DmMet was linearized by *Bst*BI digestion and integrated into the *ura3* locus in the yeast genome by homologous recombination (Fig. 1). Transformants were selected on SCD‐TRP/LEU/URA agar plates. In order to establish reference strains without Met expression, the empty plasmid pUdp6 was linearized by *Eco*RV digestion and integrated into W303a, as described above. To obtain another reference strain that does not express Tai, the empty plasmid pESC‐Leu was used in the first transformation experiment instead of pESC‐Leu‐DmTai.

### Measurement of JH/JHA activity using yeast RGA

The yeast RGA was performed as previously described [42, 44]. Single colonies of yeast strains were grown in SDC‐TRP/LEU/URA medium at 30 °C overnight, and the optical density (OD) at 595 nm of each culture was adjusted to 1.0 with the same medium. A 1‐μL aliquot of the test chemicals dissolved in DMSO, a 10‐μL aliquot of yeast cultured overnight, and 90 μL of SGC‐TRP/LEU/URA (to induce Met and Tai expression) were mixed in a 96‐well polystyrene microplate and then incubated at 30 °C for 18 h. Each cell suspension (10 μL) was transferred to a new 96‐well microplate and 100 μL of Z‐buffer (60 mm Na_2_HPO_4_, 40 mm NaH_2_PO_4_, 1 mm MgSO_4_, 10 mm KCl, 2 mm DTT, and 0.2% sarcosyl, adjusted to pH 7.0) containing 1 mg·mL^−1^ ONPG was then added and followed by an incubation at 37 °C for 60 min. Absorbance at wavelengths of 405 and 595 nm was measured using Micro Plate Reader Model 680 (Bio‐Rad Laboratories, Inc.: Hercules, CA, USA) to estimate β‐galactosidase activity as the amount of *o*‐nitrophenol (ONP) produced and yeast cell density, respectively. Juvenoid‐dependent *lacZ* reporter induction was demonstrated as an ‘increase of induction’, which was calculated using the following formula: [OD_405_ (sample)/OD_595_ (sample)] – [OD_405_ (DMSO)/OD_595_ (DMSO)]. Relative cell growth rate (% control) was calculated using the formula: [OD_595_ (18 h) – OD_595_ (0 h) in exposed wells]/[OD_595_ (18 h) – OD_595_ (0 h) in control wells]. Based on the dose–response curves of the RGA, the 50% effective concentration [EC_50_ (μm)] for each compound was evaluated using Probit transformation [51]. Data were analyzed using Student’s *t*‐test to assess the significance of differences between two sets of values. Probability (*P*) values < 0.05 were considered to be significant.

## Results

### Optimization of the yeast RGA for Met

**Fig. 2:**
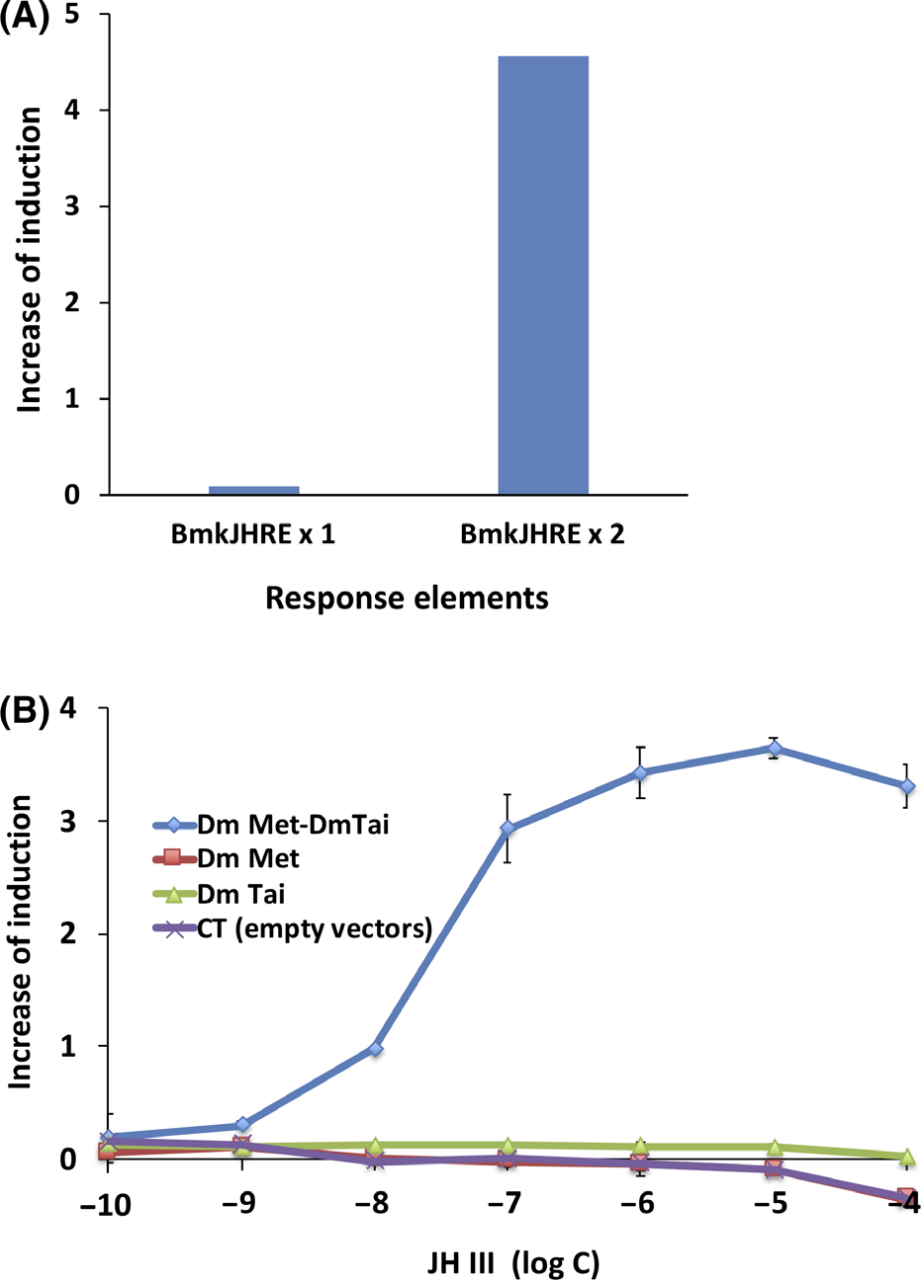
Optimization of the yeast RGA for Met. (A) Comparison of copy number of BmkJHRE in yeast RGA for Met–Tai. Met–Tai assay yeast strains were established using reporter plasmids carrying one or two copies of BmkJHRE and exposed to 0.1 μm of JH III. Data represent the average of duplicate experiments for representative yeast transformants. (B) Requirement of Tai expression for ligand responses in yeast strains expressing Met. The response to JH III in the yeast strain co‐expressing Met and Tai was compared with those expressing Met or Tai alone. A yeast strain carrying empty vectors instead of Met and Tai expression plasmids was also established as a reference. Data represent the mean ± standard deviation (SD) of triplicate experiments.

To optimize the yeast RGA for Met, we constructed new reporter plasmids carrying one or two copies of the E‐box (CACGTG)‐containing JHRE from the *B*. *mori Kr‐h1* gene (BmkJHRE) [26]. These reporter plasmids were introduced into the wild‐type yeast strain W303a along with the Met and Tai expression plasmids. A yeast strain carrying two copies of BmkJHRE exhibited reporter activity in response to JH III (0.1 μm) (Fig. 2A). Therefore, we selected pYTβ‐BmkJHRE × 2 containing two copies of Bm‐kJHRE as a reporter plasmid for the Met–Tai RGA in the present study.

We then compared the ligand responses of yeast strains expressing Met–Tai, Met, and Tai, respectively. Only the yeast strain co‐expressing Met and Tai responded to JH III and induced *lacZ* reporter gene expression. Neither of the strains expressing Met or Tai alone responded to JH III or a reference strain carrying empty plasmids instead of the Met and Tai expression plasmids (Fig. 2B).

### Responses of Met–Tai assay yeast to natural JHs

**Fig. 3:**
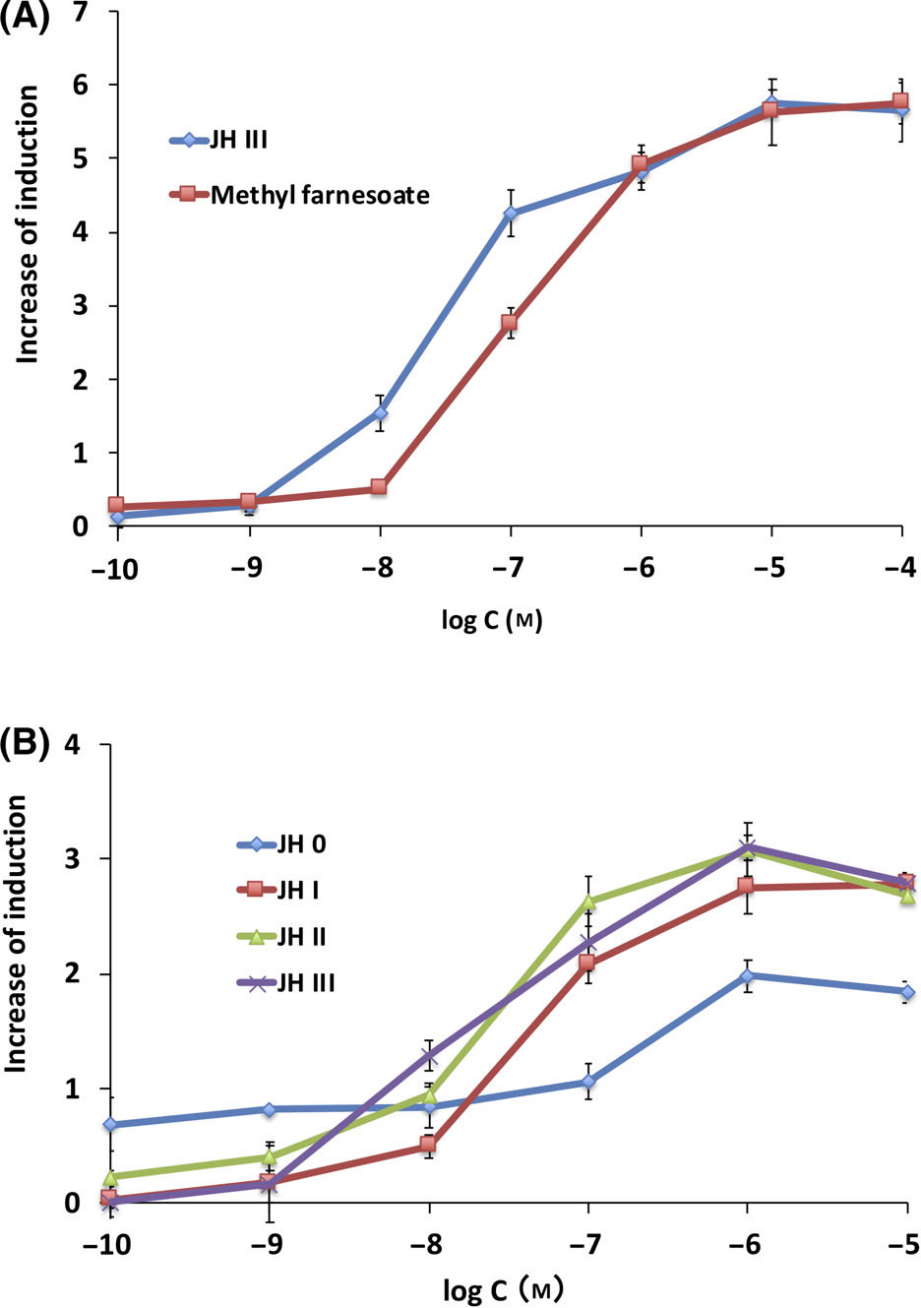
Responses to natural JHs in the Met–Tai assay yeast strain. Yeast RGA was conducted using (A) the commercially available natural JHs, JH III and methyl farnesoate and (B) in‐house synthesized JHs, JH 0, JH I, and JH II as ligands. Commercial JH III was also used as a reference. Data represent the mean ± SD of triplicate experiments.

We initially investigated the responses of the Met–Tai assay yeast strain to the commercially available natural JHs, JH III, and methyl farnesoate. As shown in Fig. 3A, Met–Tai assay yeast responded to these JHs in a dose‐dependent manner. Both compounds induced reporter gene expression at 0.01 μm (Table 1). We also used in‐house synthesized natural JHs [46] in the yeast RGA. JH I and II activated Met at 0.01 μm, and the β‐gal level increased in a dose‐dependent manner, as well as JH III (Fig. 3B, Table 2). Met–Tai assay yeast did not exhibit a dose‐dependent response up to 0.01 μm in the assay of JH 0; however, β‐gal activity was higher than that of the solvent control. JH 0 slightly potentiated reporter gene expression at high doses (Fig. 3B). The EC_50_ values of natural JHs calculated from dose–response curves were summarized in Tables 1 and 2. The EC_50_ value of JH III was approximately 2.5‐fold lower than that of methyl farnesoate, and the difference was significant (*P* < 0.001) (Table 1). Regarding in‐house synthesized natural JHs, JH 0 was a less potent ligand than other JHs (3.3‐ to 4.3‐fold difference, *P* < 0.001 for JH 0 vs. JH I or III, *P* < 0.005 for JH 0 *vs*. JH II, Table 2). No significant differences were observed in EC_50_ values among JH I, II, and III (*P* = 0.27 ˜ 0.52). The rank order of the ligand potencies of natural JHs in yeast RGA was JH II ≥ JH III ≥ JH I > methyl farnesoate > JH 0.

**Table 1:** EC_50_ values of JH III and methylfarnesoate in yeast expressing Met–Tai.

Ligand	Detection limit (μm)	EC_50_ (μm)
JH III	0.01	0.062 ± 0.005
Methyl farnesoate	0.01	0.16 ± 0.007*

* The EC_50_ value of methyl farnesoate was significantly different from that of JH III (*P* < 0.001).

**Table 2:** EC_50_ values of in‐house synthesized JHs in yeast expressing Met–Tai

Ligand	Detection limit (μm)	EC_50_ (μm)
JH 0	0.1	0.213 ± 0.014
JH I	0.01	0.065 ± 0.011*
JH II	0.01	0.050 ± 0.017**
JH III	0.01	0.057 ± 0.007*

* The EC_50_ value of JH 0 was significantly different from those of other ligands: *JH 0 *vs*. JH I or JH III (*P* < 0.001); **JH 0 *vs*. JH II (*P* < 0.005).

### Responses of Met–Tai assay yeast to synthetic JHAs

**Fig. 4:**
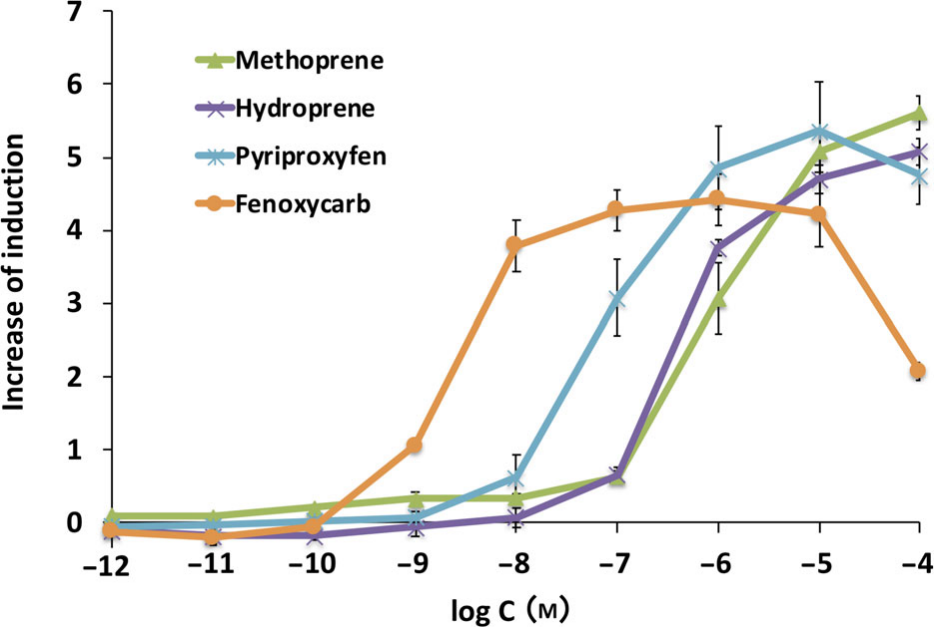
Responses to synthetic JHAs in the Met–Tai assay yeast strain. Yeast RGA was conducted using the synthetic JHAs, methoprene, (*S*)‐hydroprene, pyriproxyfen, and fenoxycarb as ligands. Data represent the mean ± SD of triplicate experiments.

We performed the yeast RGA using the synthetic JHAs, methoprene, (*S*)‐hydroprene, pyriproxyfen, and fenoxycarb as ligands. Met–Tai assay yeast responded to these ligands in a dose‐dependent manner. Fenoxycarb reduced β‐gal activity at the highest concentration due to its cytotoxicity to yeast cells (Fig. 4). Relative cell growth rate decreased to approximately 40% after 18 h exposure to fenoxycarb at 100 μm, while over 80% of the cells survived in the assays with other JHAs. Significant differences were observed in the EC_50_ values of these compounds (Table 3), and the rank order of synthetic JHAs in yeast RGA was fenoxycarb >> pyriproxyfen >> (*S*)‐hydroprene > methoprene.

**Table 3:** EC_50_ values of synthetic JHAs in yeast expressing Met–Tai

Ligand	Detection limit (μm)	EC_50_ (μm)
Methoprene	0.1	1.1 ± 0.23
(*S*)‐hydroprene	0.1	0.57 ± 0.15^*^
Pyriproxyfen	0.01	0.087 ± 0.012**^,^^#^
Fenoxycarb	0.001	0.003 ± 0.001**^,^^##, $^

The EC_50_ values of synthetic JHAs were significantly different from each other: *methoprene *vs*. (*S*)‐hydroprene (*P* < 0.05); **methoprene *vs*. pyriproxyfen or fenoxycarb (*P* < 0.005); ^#^(*S*)‐hydroprene *vs*. pyriproxyfen (*P* < 0.01); ^##^(*S*)‐hydroprene *vs*. fenoxycarb (*P* < 0.005); ^$^pyriproxyfen *vs*. fenoxycarb (*P* < 0.001).

### Relationships between ligand potencies of juvenoids among RGAs for Met–Tai established in yeast and animal cells

**Fig. 5:**
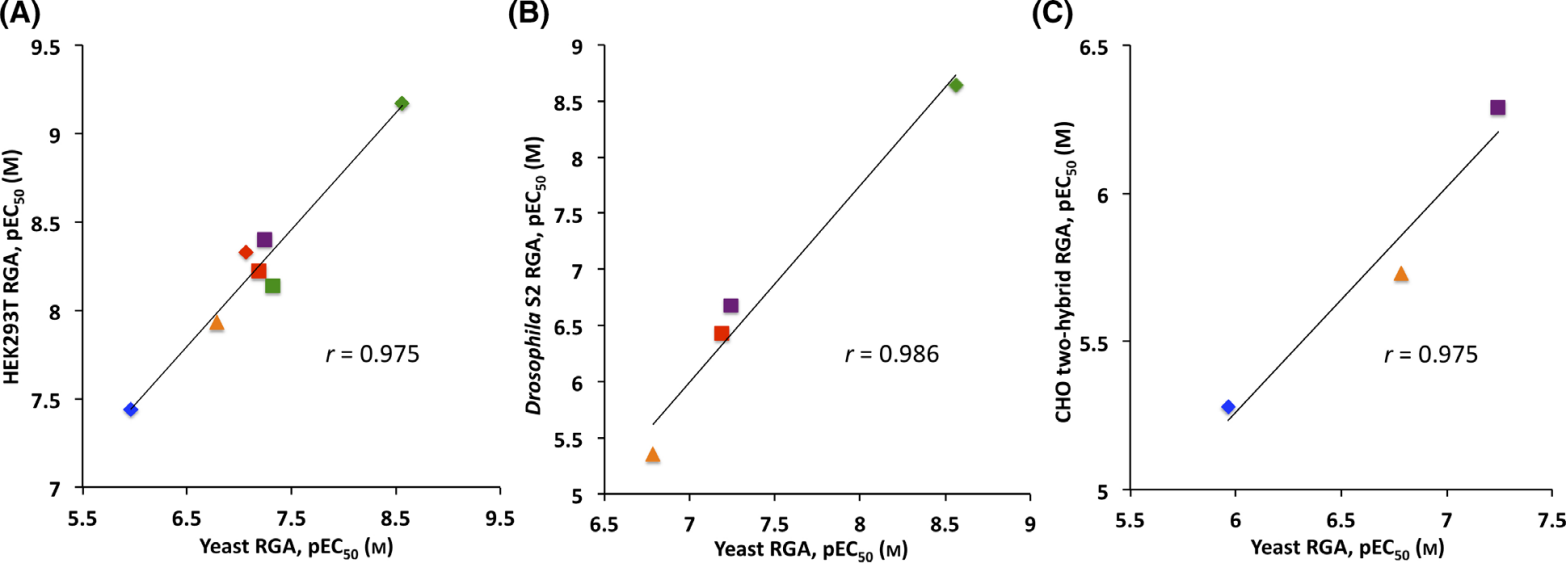
Relationships between ligand potencies of natural JHs and synthetic JHAs among the yeast RGA and previously established RGAs in animal cells (pEC_50_). (A) Comparison with the HEK293T RGA [46]. (B) Comparison with the RGA in *Drosophila* S2 cells expressing endogenous JHRs [38]. (C) Comparison with the two‐hybrid‐based RGA established in CHO cells [48]. Natural JHs; JH I (red square), JH II (green square), and JH III (purple square), and methyl farnesoate (orange triangle). Synthetic JHAs: methoprene (blue diamond), pyriproxyfen (red diamond), and fenoxycarb (green diamond). Correlation coefficients (*r*) are shown in each graph.

Three RGAs for Met–Tai were recently established in insect and mammalian cell systems. As a mammalian RGA cell system, the expression plasmids for Met and Tai and the luciferase reporter plasmid under the control of BmkJHRE were introduced into HEK293T cells derived from human embryonic kidney cells [46]. To establish a RGA in insect cells, Bittova *et al*. introduced a luciferase plasmid under the control of the JHRE of the *A*. *aedes* early trypsin (Aa ET) gene into *D. melanogaster* Schneider 2 (S2) cells expressing endogenous JH receptors [38]. A two‐hybrid‐based RGA in which Met and Tai were fused to the VP16 activation domain (VP16 AD) and GAL4 DNA‐binding domain (GAL4 DBD), respectively, were also established in Chinese hamster ovary (CHO) cells [48]. We compared the potencies of natural JHs and synthetic JHAs between yeast and RGAs established in HEK293T, *Drosophila* S2, and CHO cells by plotting pEC_50_ values, the negative logarithms of EC_50_ (Fig. 5). The sensitivity of yeast RGA was 2‐ to 30‐fold lower than that of the HEK293T RGA. On the other hand, yeast RGA was more sensitive than the *Drosophila* S2 RGA and CHO two‐hybrid RGA: ligand potencies in the yeast RGA were 4‐ to 27‐fold and 5‐ to 11‐fold higher than those in the *Drosophila* S2 and CHO two‐hybrid RGAs, respectively. The correlation coefficient (*r*) of regression lines between yeast and other RGAs were high, with *r* values of 0.975–0.986 (Fig. 5). This result indicates that the rank order of the ligand potencies of test compounds strongly correlated among yeast, mammalian, and insect cell RGA systems (Fig. 5).

## Discussion

RGA is a widely used experimental method that has been employed to examine the activity of synthetic chemicals that mimic the MHs and JHs of insects. These insect hormones exert their effects by activating the specific receptors EcR–USP and Met for MHs and JHs, respectively. EcR–USP and Met are ligand‐dependent transcription factors that belong to the nuclear receptor superfamily and bHLH‐PAS domain protein family, respectively (reviewed by Ito‐Harashima and Yagi [39]). We previously established RGA systems for insect EcR–USP in the yeast *S*. *cerevisiae*, in which EcRs and USPs from three different taxonomic orders were introduced in conjunction with the steroid receptor coactivator Tai and reporter plasmids containing appropriate response elements [44]. *S*. *cerevisiae* is the simplest eukaryote possessing highly conserved gene expression systems with those of animals, including insects and humans [52]. The mechanisms of action and ligand responses of EcR–USP from three insect species were reconstituted well in yeast RGAs [44].

In insect cells, the functional JH receptor is constituted by the heterodimer of Met and Tai proteins [23, 24, 25]. In the present study, we newly established a yeast‐based RGA to detect JHs/JHAs by introducing cDNA fragments containing full‐length *D. melanogaster* Met and Tai ORFs (Fig. 1). We showed that the E‐box‐containing JHRE identified in the *B*. *mori Kr‐h1* gene (BmkJHRE) [26] was effective for JH III‐dependent gene expression in the yeast RGA (Fig. 2A). This result suggests that the DNA‐binding properties of Met proteins were conserved among the different insect species because of the high sequence similarity for the bHLH domain involved in DNA binding [23, 25, 38]. We also demonstrated that JH III‐dependent reporter gene expression was only observed in the yeast strain co‐expressing Met and Tai (Fig. 2B), suggesting that the heterodimerization of Met and Tai was required for ligand‐dependent transactivation in yeast as well as insect cells [23, 24, 25].

We examined whether the established yeast RGA for Met–Tai was responsive to known juvenoids. As shown in Figs. 3 and 4, Met–Tai assay yeast exhibited dose‐dependent responses to natural JHs and synthetic JHAs. Differences in ligand potencies among juvenoids, particularly synthetic JHAs, were clearly detectable (Tables 1, 2, 3). The sensitivity of the yeast RGA was lower than that of the HEK293T RGA, but higher than RGAs established in *Drosophila* S2 and CHO cells. To improve the sensitivity of the yeast RGA, increases in the copy number of BmkJHRE or the use of JHRE of the *D. melanogaster Kr‐h1* gene [53] may be effective. Alternatively, the deletion of the genes encoding cell wall mannoproteins (*CWP1*/*CWP2*) and/or plasma membrane efflux pumps (*PDR5*/*PDR10*), or *ERG6*, which is involved in the synthesis of plant/fungal‐specific ergosterol, may improve the permeability of the cell wall/plasma membrane and increase the intracellular concentrations of test compounds [42, 43, 54, 55, 56]. We also showed that the rank order of ligand potencies in the yeast RGA strongly correlated with those obtained in the other RGA systems (Fig. 5). JH III is the most common in various insects while unique types of JHs have been found in some insect orders [9, 57]. JH III bisepoxide (JHB_3_) was identified as a JH specific to higher Dipteran insects including *D. melanogaster* [58, 59], and its agonist potency for *Drosophila* JH receptors was indicated *in vitro* [38]. It is important to evaluate the effectiveness of our yeast RGA for Met–Tai in detecting JHB_3_ compared to other JHs. It is also interesting to establish JH‐type specific RGAs in the future. Two‐hybrid‐based yeast RGAs to detect interactions between *A*. *aedes* Met and Tai orthologs were recently established and utilized for the screening of plant compounds with JH/anti‐JH activity [60, 61, 62]. We have previously shown that the yeast RGAs for human nuclear receptors detected not only agonist but also antagonist activities of various ligands [42, 43, 47]. Our newly established yeast RGA for Met–Tai is useful for screening more potent JH/anti‐JH‐like compounds from a pool of natural and synthetic chemical compounds.

In the present study, the JH‐Met signaling pathway for the transcriptional activation of target genes was reconstituted *via B. mori Kr‐h1* JHRE in the yeast strain co‐expressing Met and Tai. This procedure is simple and may be applied to the establishment of new yeast RGAs for Met from other insect and arthropod species. Further studies are warranted to examine whether species‐selective responses to JHs/JHAs exist. In contrast to synthetic MH agonists, JHAs with potent insect selectivity have not yet been discovered [63]. Extensive efforts have recently been made to obtain a novel class of JHAs using combined approaches, such as (quantitative) structure‐activity relationship ((Q)SAR) studies, virtual screening methods, including the three‐dimensional (3D) modeling of the ligand‐binding pocket and ligand docking studies, and RGA methods [46, 64, 65]. The yeast RGA is available as an activity‐based high throughput screening method for the compounds identified in (Q)SAR and/or virtual screening approaches. Yeast‐based RGA systems for the insect hormone receptors, Met–Tai (this study) and EcR–USP of three different taxonomic orders [44], are rapid, easy to handle, and cost‐effective experimental methods, and valuable as primary screening tools for detecting new IGRs with MH‐ and JH‐like activities.

## Conflict of interest

The authors declare no conflict of interest.

## Author contributions

SIH designed the research project. MM, SIH, and ET performed experiments and analyzed data. SIH conducted data interpretation and wrote the manuscript. MK contributed to the preparation of essential materials and resources from the grants. YN provided in‐house synthesized JHs and made comments on the experiments throughout the study. TY organized the research project, reviewed the manuscript, and arranged necessary resources from the grants. All authors approved the final version of the manuscript.

## Supporting information

**Table S1**. Primer sequences.

## Data Availability

The data that support the finding of this study are available in figures, tables, and the supplementary material of this article.
