# Supplementary material for: Detection of juvenile hormone agonists by a new reporter gene assay using yeast expressing Drosophila methoprene‐tolerant
Source: FEBS Open Bio. 2021 Aug 30;11(10):2774–83. doi: 10.1002/2211-5463.13277 (PMC8487040; doi:10.1002/2211-5463.13277)
Supplement: Supplementary file 1 — Table S1. Primer sequences. [file FEB4-11-2774-s001.docx]

Table S-1. Primer sequences

| Primer | Sequence (5’-3’) | Comments |
| --- | --- | --- |
| DmMet-Fwd^a, b^ | CCCCCCCGGG*AACAAA*ATGGCAGCACCAGAGACGGGCAAC | Amplify DmMet ORF |
| DmMet-Rev^a^ | CCCCGAATTCTCATCGCAGCGTGCTGGTCAGGGT | Amplify DmMet ORF |
| Bm-kJHRE Fwd^c^ | CTAGTAAACCGCGGTGGGCCTCCACGTGTCGAACGCTACCGCTTGCCCCACCCCATCTCCTTCACACCGCAT | Response element |
| Bm-kJHRE Rev^c^ | CTAGATGCGGTGTGAAGGAGATGGGGTGGGGCAAGCGGTAGCGTTCGACACGTGGAGGCCCACCCCGGTTTA | Response element |

^a^Underlined sequences are additional nucleotides to introduce restriction enzyme recognition sites

^b^The sequences shown in italics are yeast ribosomal-binding consensus sequences

^c^JHRE sequences for *B. mori* Met were underlined.
